# Reagentless Sensing of Vancomycin Using an Indium Tin Oxide Electrode Grafted with Molecularly Imprinted Polymer including Ferrocenyl Group

**DOI:** 10.3390/s21248338

**Published:** 2021-12-13

**Authors:** Haruto Eguchi, Akihiko Hatano, Yasuo Yoshimi

**Affiliations:** 1 Department of Applied Chemistry, Shibaura Institute of Technology, Tokyo 135-8548, Japan; mc18007@shibaura-it.ac.jp; 2 Department of Material Engineering, Shibaura Institute of Technology, Saitama 337-8570, Japan; a-hatano@sic.shibaura-it.ac.jp; 3 Innovative Global Program, Shibaura Institute of Technology, Tokyo 135-8548, Japan; 4 Japanese Association of Bio-Intelligence for Well-Being, Saitama 337-8570, Japan

**Keywords:** molecularly imprinted polymer, vancomycin, therapeutic drug monitoring, allylamine carboxypropionate-3-ferrocene, differential pulse voltammetry, sensor, indium tin oxide, blood, graft polymerization

## Abstract

Vancomycin (VCM) is a first-line antimicrobial agent against methicillin-resistant Staphylococcus aureus, a cause of nosocomial infections. Therapeutic drug monitoring is strongly recommended for VCM-based chemotherapy. The authors attempted to develop a simple VCM sensor based on molecularly imprinted polymer (MIP), which can be used with simple operations. Methacrylic acid (MAA), acrylamide, methylenebisacrylamide, and allylamine carboxypropionate-3-ferrocene (ACPF) were copolymerized in the presence of VCM and grafted from the surface of indium-tin oxide (ITO) to obtain MIP-coated electrodes. The MIP-grafted ITO electrode was used for differential pulse voltammetry (DPV) measurements in a buffer solution containing VCM or whole bovine blood. The obtained current depends on the VCM concentration with high linearity. The dynamic range covered the therapeutic range (20–40 μg/mL) of the VCM but was almost insensitive to teicoplanin, which has a similar structure to VCM. The ITO electrodes grafted by the same procedure except for omitting either VCM or APCF were not sensitive to VCM. The sensitivity of the MIP electrodes to VCM in whole blood and buffered saline, but the background current in blood was higher than that in saline. This high background current was also seen in the deproteinized plasma. Thus, the current is probably originated from the oxidation of low molecular weight reducing agents in the blood. The MIP-grafted ITO electrode using ACPF as a functional monomer would be a promising highly selective sensor for real-time monitoring of VCM with proper correction of the background current.

## 1. Introduction

Vancomycin (VCM) is the first line of an antibacterial agent against methicillin-resistant *Staphylococcus aureus* (MRSA), a major nosocomial pathogen [1]. Overdose of VCM induces nephrotoxicity or ototoxicity, while underdose allows the creation of VCM resistant bacteria, which is a problem over the world [2,3,4,5,6,7]. Therefore, therapeutic drug monitoring (TDM) is strongly recommended for chemotherapy with VCM [1]. TDM is the individualized dosage and administration of drugs such as VCM to each patient while monitoring for factors related to therapeutic effects and side effects. Current TDM for VCM generally uses immunoassay [8,9,10], for which procedures require complicated techniques, such as pipetting, incubation, and centrifugation. The hospitals must outsource the analysis to an external agency that takes 2–3 days to obtain the measurement data. Therefore, there is a need for the development of sensors that can measure VCM concentrations in real-time. The purpose of this study is the development of sensors that can quickly obtain data on VCM concentrations in blood at the bedside with simple manipulation to realize the real-time monitoring of VCM in blood. A sensor that can detect VCM in the blood directly from whole blood without any pretreatment, such as the blood glucose sensors currently available on the market [11,12,13], would be able to achieve this goal.

Therefore, we attempted to develop an electrochemical VCM sensor using a molecularly imprinted polymer (MIP) grafted on an electrode. MIP is a polymer with a specific binding capacity to target material and can be synthesized by copolymerizing cross-linking monomer and functional monomer in the presence of the target material as a template. MIP can be prepared at low cost and with simple manufacturing. Thus, many researchers have attempted to develop sensors taking advantage of MIPs [14,15,16]. Especially, electrochemical sensors based on MIPs are expected to have a wide range of applications due to the simple structure of the devices for electrochemical analysis [16]. Recently, electrochemical sensors based on MIPs have been actively developed for clinical applications [17,18,19]. Once the MIP-based electrochemical VCM sensor is completed, it is expected to be readily available in developing countries where the epidemic of resistant bacteria is becoming more serious. Mazzotta et al. have developed an amperometric sensor consisting of nanoparticles of VCM-MIP containing ferrocenyl groups immobilized on the surface of a glassy carbon electrode [20]. Since the redox current of the ferrocenyl group is detected by cyclic voltammetry, simple reagentless sensing is possible without the need for the addition of any indicators. Unfortunately, however, the sensor was not sensitive enough to measure the blood concentration of VCM.

The novelty of this work is the development of a sensor for VCM by coating MIP on the surface of indium tin oxide (ITO) covalently by graft polymerization. We previously succeeded in creating MIP electrodes by grafting the MIP layer onto the surface of the ITO where the photo-initiator of radical polymerization is coated through a simple procedure [21,22,23]. ITO is a commonly used material for liquid crystal displays and is cheaply available, thus the MIP-grafted electrodes are feasible as inexpensive disposable sensors enabling simple operation and responding quickly: e.g., the response time of the MIP-electrode of heparin was less than 20 s [22]. The property of the MIP-grafted electrode is promising for a disposable sensor for real-time analysis.

In this study, we created a VCM sensor by grafting MIP on the initiator-coated ITO base electrode, which generates current responding to the concentration of VCM. We also selected the most suitable ferrocenyl monomer for this sensor and further evaluated its performance as a sensor for VCM in whole blood.

## 2. Experiment

### 2.1. Chemicals

3-Aminopropyltrimethoxysilane was purchased from Tokyo Kasei Co., Ltd. (Tokyo, Japan). VCM, p-chloromethylbenzoic acid, sodium diethyldithiocarbamate trihydrate acrylamide, *N*,*N*-methylenebisacrylamide (MBAA), vinyl ferrocene (VF), and methacrylic acid were purchased from Wako Pure Chemical Co., Ltd. (Osaka, Japan). Potassium hydroxide, acetone, methanol, *N*,*N*-dimethylformamide (DMF), Toluene, and ethanol were purchased from Kanto Chemical Co., Ltd. (Tokyo, Japan), and 1-ethyl-3-(3-dimethylaminopropyl) carbodiimide hydrochloride (water-soluble carbodiimide) was purchased from Dojindo Laboratories (Kumamoto, Japan). Indium-tin oxide (ITO) sputtered on a glass plate was purchased from Furuuchi Chemical Co. (Tokyo, Japan). Quartz plates were purchased from Monotech, Inc. (Saitama, Japan). Ferrocenyl methyl methacrylate was purchased from Shanghai UCHEM Inc. (Shanghai, China). Bovine blood (including 5 g/L sodium citrate as an anticoagulant) was purchased from Tokyo Shibaura Zoki Inc. (Tokyo, Japan).

Allylamine carboxypropionic-3-ferrocene (ACPF) was synthesized by the procedure published previously [24].

### 2.2. Graft Polymerization of MIP from ITO Electrode

**Figure 1:**
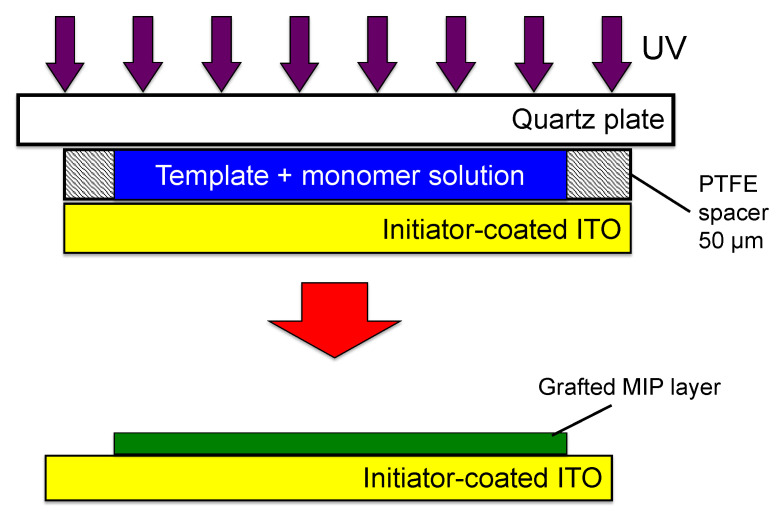
Scheme of the procedure of MIP grafting on the initiator-coated ITO by UV irradiation.

ITO electrode (10 cm × 10 cm) was cut with a diamond cutter into an area of 3.0 cm × 5.0 cm. Diethyldithiocarbamate methylene group, which works as a photo-initiator of radical polymerization, was introduced on the surface of the ITO electrode by the procedure described previously [22]. VCM (0.08 mmol, 0.120 g), methacrylic acid (4.2 mmol, 0.360 g), acrylamide (5.1 mmol, 0.360 g) and 0.100 g ferrocenyl monomer (VF (0.47 mmol), ACPF (0.30 mmol) or FMMA (0.35 mmol)), methylenebisacrylamide (6.5 mmol, 1.000 g) were dissolved in 7.5 mL of a mixed solvent (1.5 mL of distilled water, 6.0 mL of DMF). The solution was bubbled with argon (20 min). The solution was sandwiched between the initiator-coated ITO electrode (3.0 or 4.0 cm × 5.0 cm) and the quartz crystal plate (Figure 1) in a glove box filled with argon. The sandwich was irradiated with a high-pressure mercury lamp (Sen Special Light Source Co., Ltd., HB100A-1, Osaka, Japan) at room temperature for 1 h. After the irradiation, the ITO was ultrasonicated in distilled water (10 min × 3 times) to remove the bulk polymer, and then immerse in 1 M NaCl aqueous solution (200 mL) for 15 min. Thereafter, the ITO electrode was washed with distilled water. The ITO electrode from which the template was extracted was cut into 1.0 cm × 5.0 cm. A non-imprinted polymer (NIP) was also prepared using the same procedure except for omitting the VCM template. The elemental compositions of the surfaces of treated and untreated ITO specimens were analyzed by X-ray photoelectron spectroscopy (XPS) using a Kratos Axis-Ultra DLD (Shimadzu Co., Ltd., Kyoto, Japan). The area of peaks of photoelectrons from indium (3d), iron (2p), tin (3d), carbon (1s), oxygen (1s), and nitrogen (1s) were evaluated.

### 2.3. Electrochemical Sensing

The working electrode was the treated or untreated ITO. The counter electrode was a platinum wire, and the reference electrode was an Ag/AgCl electrode RE-1B (BAS Co., Ltd., Tokyo, Japan). Those electrodes were installed in a miniature electrochemical cell (Plate Material Evaluating Cell, ALS Co., Ltd., Tokyo, Japan) with an internal volume of approximately 1.2 mL. The cell fixed the effective surface area of the working electrode 0.478 cm^2^. Those electrodes were connected to potentiostat (HZ-7000 Hokuto Denko, Tokyo, Japan). Samples containing 0–40 µM VCM in phosphate buffer saline (PBS: containing 0.1 M NaCl, 0.05 M sodium phosphate of pH 7.4) or in bovine whole blood. Differential pulse voltammetry (DPV) was performed. The pulse potential height was 100 mV, the potential step period was 1000 ms, the step potential height was 10 mV, the pulse width was 100 ms, and the potential scanning range was set to −0.20 to 0.90 V. The MIP electrode was immersed in 1 M NaCl aqueous solution and washed to remove the template and rinsed with distilled water and dried by blowing with nitrogen gas, between each measurement.

## 3. Results and Discussion

### 3.1. Characterization of VCM-MIP Electrodes

**Figure 2:**
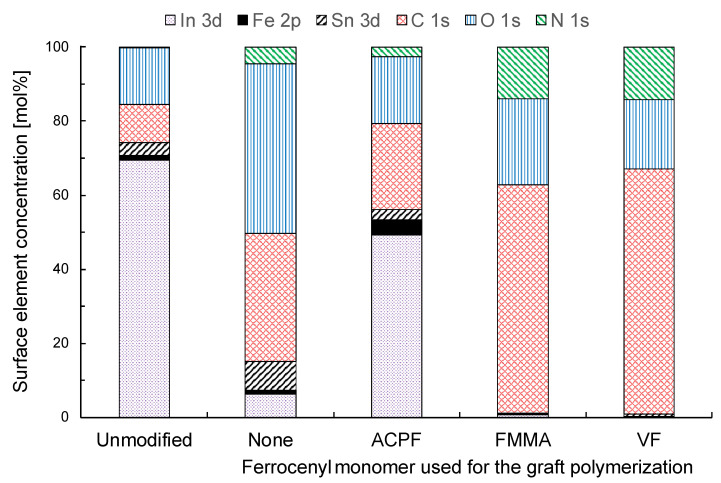
Elemental composition of each electrode surface.

The elemental composition of the surfaces of the modified and unmodified ITO calculated by XPS analysis is shown Figure 2. All the grafted ITO samples indicated higher nitrogen concentration than nontreated ITO did. The result indicates the graft polymerization was successfully performed at the initiator coated ITO surface. Indium and tin were hardly detected at the surface of the ITO sample grafted with MIP prepared using VF or FMMA, which indicates that the thickness of the grafted layer was beyond the limit detection depth of the XPS (ca. 10 nm). In contrast, indium and tin were detected more clearly at the MIP-grafted surface using ACPF than at any other grafted samples indicating that the thickness of the grafted layer is the thinnest. The property of the ACPF-including MIP electrode is similar to the MIP-grafted electrodes which we previously developed [21,22]. Iron was most abundantly detected in the samples containing ACPF. Those results may indicate that ACPF suppresses the copolymerization of MAA, MBAA, and AAm, while VF and FMMA promote copolymerization. It is known that allyl compounds usually polymerize with low reaction rates because of degradative chain transfer to the monomer [25]. It may be likely that the ACPF has decelerated the chain reaction radical polymerization, similarly.

### 3.2. Sensitivity of MIP-Grafted Electrode

**Figure 3:**
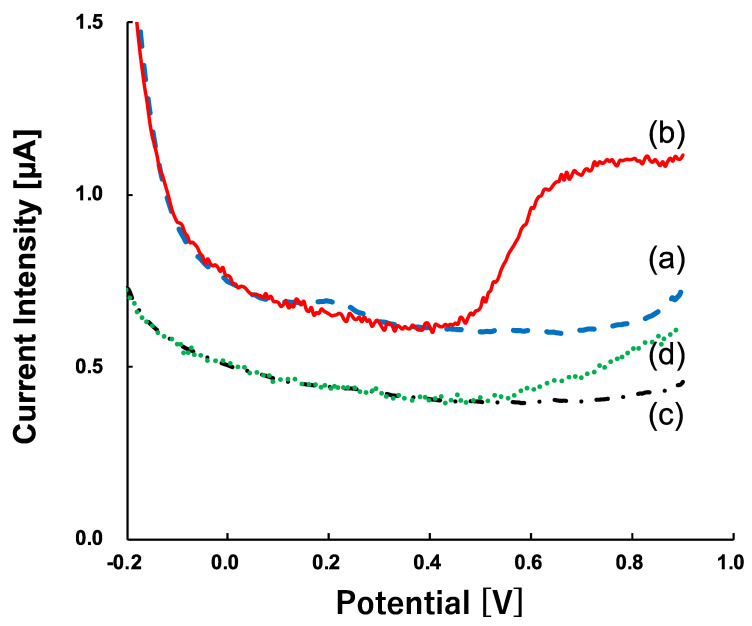
Differential pulse voltammogram with ACPF-containing VCM-MIP electrode in (**a**) 0 µM and (**b**) 40 µM VCM solution and that with unmodified ITO electrode in (**c**) 0 M and (**d**) 40 µM VCM solution.

Differential pulse voltammogram with unmodified ITO and with the MIP-grafted (ACPF was used) is shown in Figure 3. The addition of 40 µM VCM increased the currents at both electrodes above 0.5 V vs. Ag/AgCl potential. The increase of the current at the MIP-grafted electrode is clearer than that at the unmodified one. The result indicates that VCM is anodically active at the surface of the ITO electrode, and the activity is enhanced by the interaction between the VCM and the MIP on the ITO. 

**Scheme 1:**
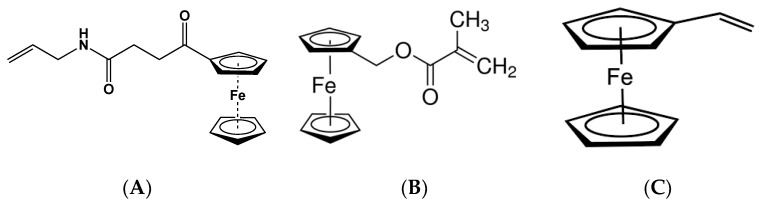
Structure of ferrocenyl monomers used in this work (**A**) ACPF, (**B**) FMMA, and (**C**) VF.

**Figure 4:**
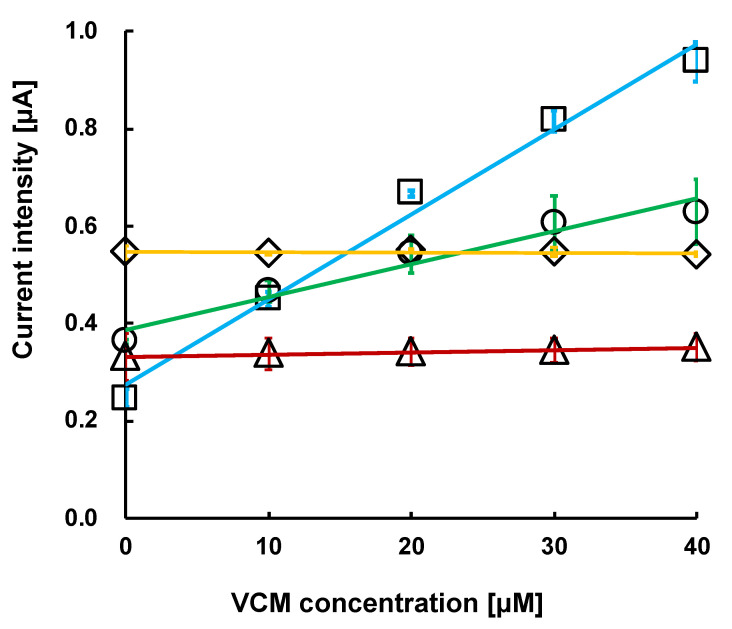
Relationship between VCM concentration and current intensity at 0.80 V vs. Ag/AgCl. ACPF (blue square), FMMA (yellow diamond), or VF (green circle) was used for the MIP synthesis. MIP electrode without ferrocenyl monomer (red triangle).

Figure 4 illustrates the relationship the detected current at 0.8 V vs. Ag/AgCl at the MIP-ITO using APCF, FMMA, VF, (Scheme 1) and none as the ferrocenyl monomer. The MIP grafted ITO without ferrocenyl group was insensitive to VCM. The sensitivity (the slope of the regression line) is listed in Table 1. The MIP including FMMA was also insensitive. Only the MIP-grafted ITO using VF or ACPF indicated sensitivity to VCM. The result indicates that the ferrocenyl group is mandatory for electrochemical sensing with the MIP-grafted electrode, but the ferrocenyl group of FMMA does not contribute to the detection of VCM. 

**Figure 5:**
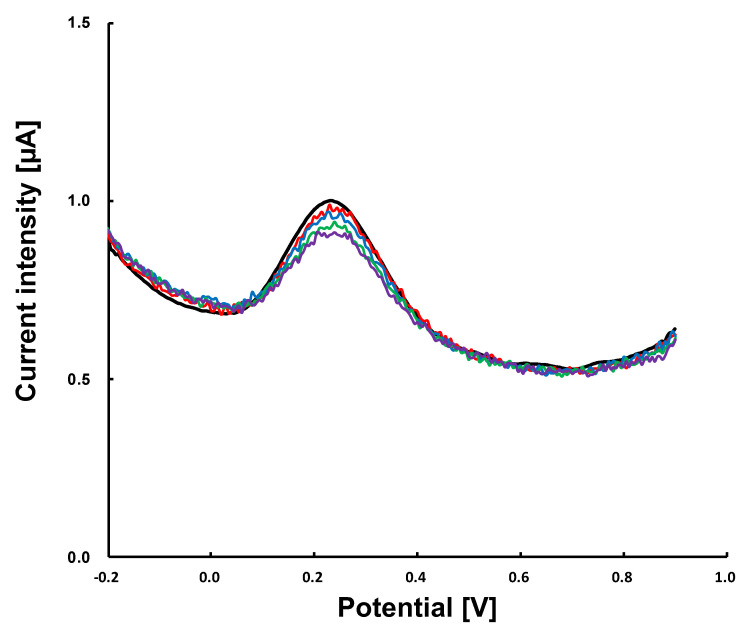
A differential pulse voltammogram at the MIP electrode using FMMA with the VCM concentration of 0 (black), 10 (red), 20 (blue), 30 (green) and 40 (purple) μM. (The peak current decreased with the number of measurements, independent of the VCM concentration).

It was somewhat surprising that the MIP using FMMA did not show the sensitivity of VCM because the result looks inconsistent with Mazzotta et al.’s work [20]. The differential pulse voltammogram of the electrode showed peak current of the ferrocenyl group around 0.25 V as shown in Figure 5, indicating that FMMA took part in the copolymerization although the XPS analysis did not detect the iron at the surface. Mazzotta et al. reported that the anodic current at a glassy carbon electrode coated with MIP-nanoparticle of VCM using FMMA decreased by the increased VCM concentration with the dynamic range of 100–400 µM [20]. This discrepancy between the two works may be because vancomycin is electrochemically inert on the glassy carbon surface, whereas it is oxidized on the ITO anode. In Mazzotta’s study, the interaction between VCM and MIP might have inhibited the electron transfer between the ferrocenyl group inside the MIP and the substrate electrode. On the other hand, the change in the current in the present study suggests that the MIP attracts VCM by specific interaction, which concentrates VCM near the electrode surface and promotes the oxidation of VCM on the ITO anode surface.

### 3.3. Sensitivity and Selectivity of VCM-MIP Electrodes

**Scheme 2:**
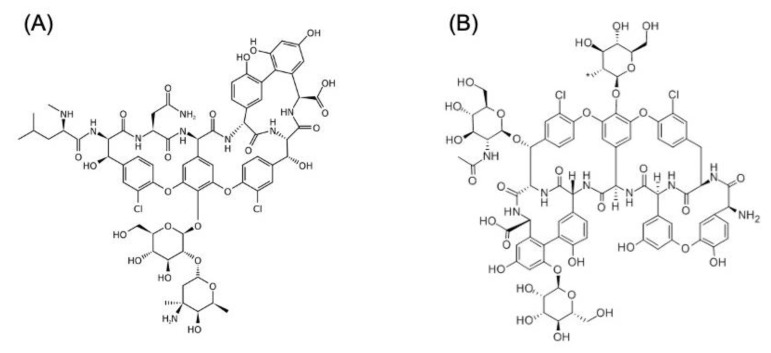
Molecular structure of VCM (**A**) and TEIC (**B**).

The molecular selectivity of VCM-MIP in buffer solution was evaluated by comparing the response to teicoplanin (TEIC), which is an antibacterial drug with similar structure as VCM as shown in Scheme 2.

**Figure 6:**
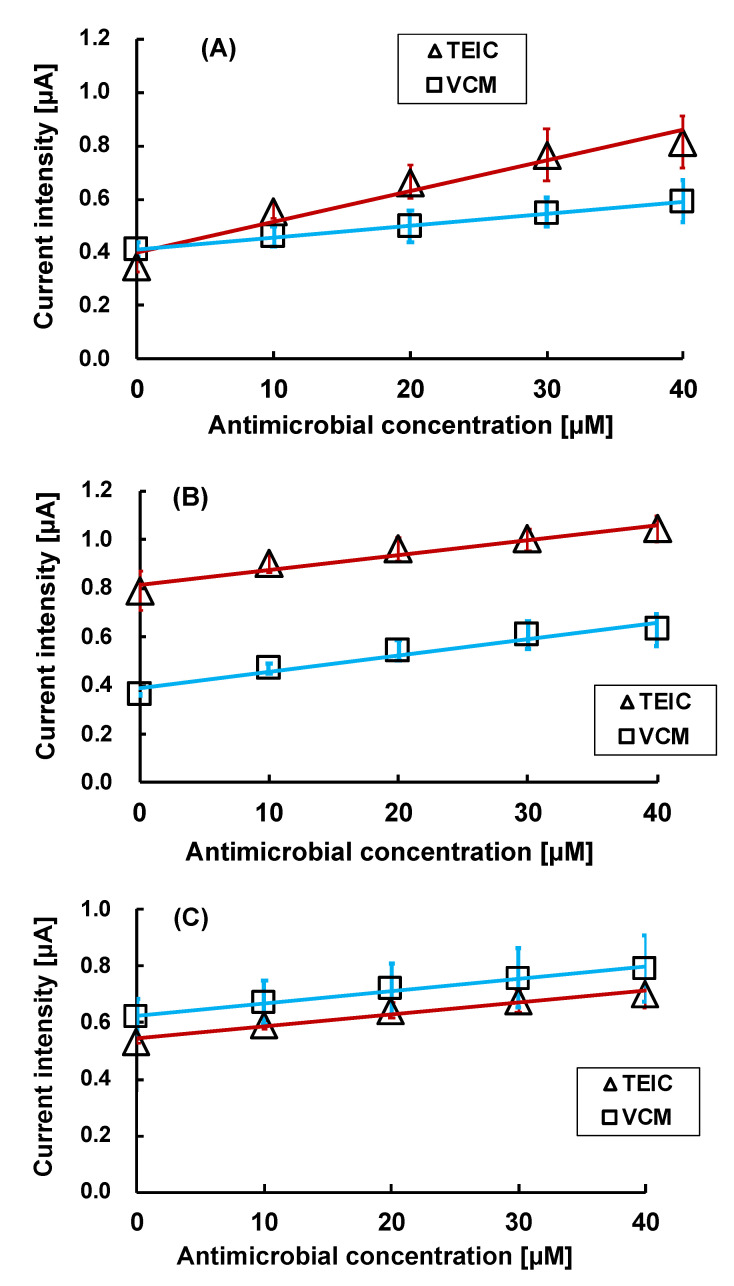

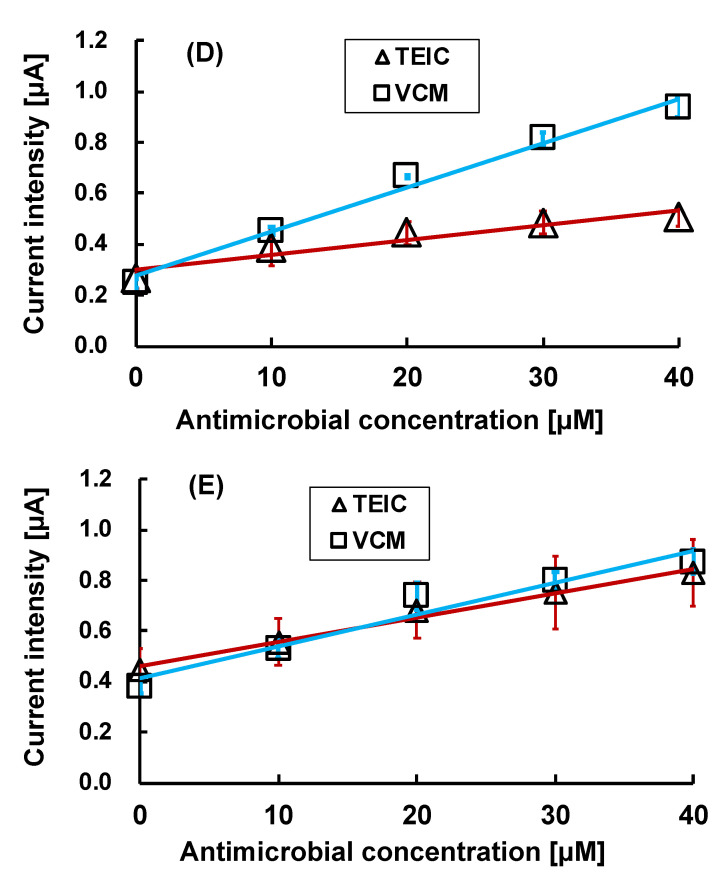
Relationship between VCM or TEIC concentration and current intensity at 0.80 V. (**A**) The unmodified electrode. (**B**) VF-MIP electrode. (**C**) VF-NIP electrode. (**D**) ACPF- MIP electrode. (**E**) ACPF-NIP electrode.

Figure 6A–E show a relationship between the current intensity at 0.80 V vs. Ag/AgCl and the antimicrobial concentration. In the concentration range 0–40 µM of VCM, all relationships showed high linearity (*R*^2^ > 0.900). This concentration range translates to 0–60 µg/mL, which covers the target trough range of (15–20 µg/mL) [21] for VCM blood concentration and the peak concentration range (20–40 µg/mL) [1]. The sensitivity of each electrode to antimicrobial agents is shown in Table 2.

The unmodified electrode was more sensitive to teicoplanin than VCM, which indicates that teicoplanin has higher electrochemical activity than VCM at the surface of the ITO electrode. In contrast, ACPF-MIP electrodes were more sensitive to VCM than to teicoplanin, but ACPF-NIP electrodes showed similar sensitivity to VCM and teicoplanin. VF-containing MIP indicated higher sensitivity to VCM than VF-NIP did. However, the selectivity of the VF-containing MIP was lower than that of ACPF-MIP. Those results indicate that ACPF is more suitable than VF for the preparation of highly selective and sensitive MIP-electrode to VCM.

### 3.4. Speculation of the Role of Ferrocenyl Monomer

**Figure 7:**
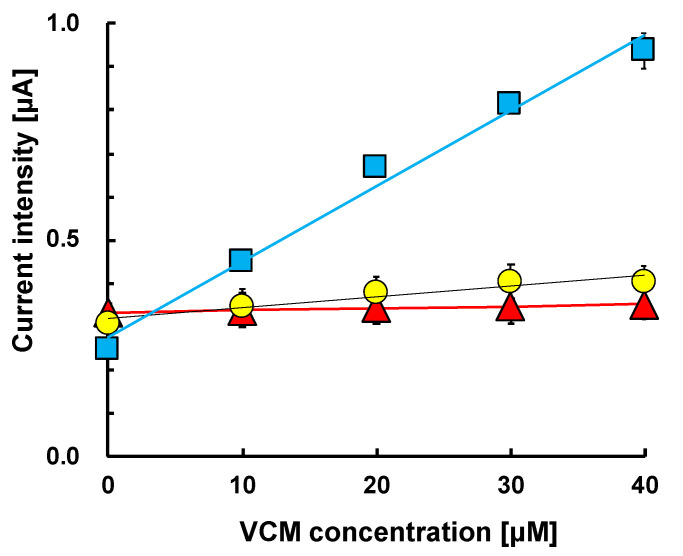
Relationship between VCM concentration and current intensity at MIP-ITO electrodes using both ACPF and MAA (squares), using ACPF only (circles), and using MAA only (triangles) during the graft-copolymerization process.

The potential required for oxidation of VCM (>0.5 V vs. Ag/AgCl) is higher than that of ferrocenyl compounds (around 0.2–0.3 V vs. Ag/AgCl). Thus, it is unlikely that the ferrocenyl group in MIP works as a mediator of electrons from VCM to the ITO base electrode. To confirm the role of the ferrocenyl group, another ACPF-MIP electrode was prepared by the same procedure except for omitting MAA and used for the DPV with the same procedure. The dependency of the current at 0.8 V vs. Ag/AgCl on the VCM concentration is shown as circle plots in Figure 7. The sensitivity to the VCM of the ACPF-MIP without MAA was 2.5 mA/M, which is drastically lower than that of ACPF-MIP with MAA, though slightly higher than the MIP with MAA but without any ferrocenyl monomer. The result suggests that ACPF works as a functional monomer that has an affinity with VCM. It also suggests that APCF, MAA, and VCM may cooperatively form a stable complex during the graft copolymerization.

**Figure 8:**
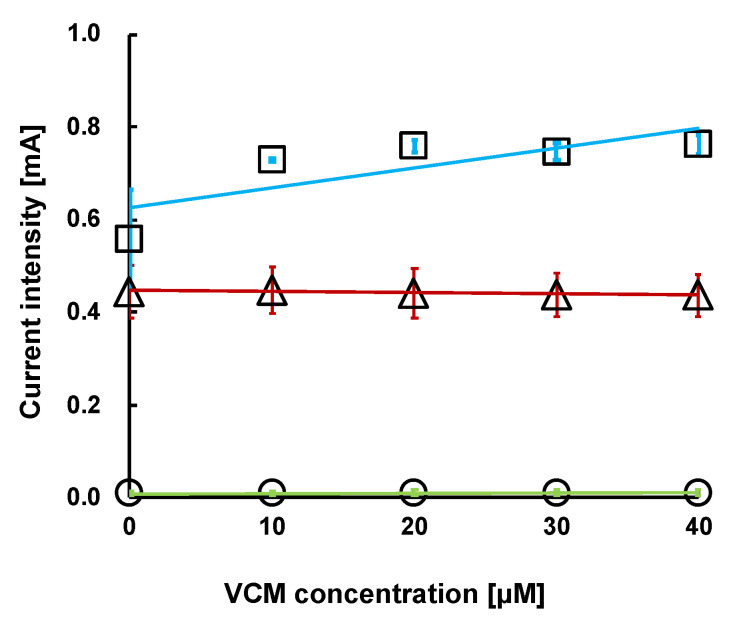
Relationship between the peak oxidative current of 5 mM ferrocyanide and VCM concentration at CV with MIP electrodes using ACPF (red triangle), that without ferrocenyl monomer (green circle) and unmodified ITO electrode (blue square).

In some previous works, we discovered that the redox current of redox marker dissolved in the sample solution at cyclic voltammetry with MIP-grafted ITO electrodes is sensitive to the concentration of the target materials used as a template. Thus, cyclic voltammetry of 5 mM potassium ferrocyanide at the electrodes of MIP-grafted ITO and untreated ITO (potential scan rate 0.2 V/s, scanning range between −0.2 and 0.8 V). The dependency of the anodic peak current of ferrocyanide on the VCM concentration is shown in Figure 8. The oxidative current was not observed at the ITO electrode grafted with MIP without ferrocenyl monomer. However, the current was detected at the MIP-grafted ITO electrode containing ACPF, but the peak current was insensitive to VCM. The results indicate that the redox marker (ferrocyanide anion) could not diffuse across layers of the MIP-without ACPF but can diffuse across the ACPF-MIP. The speculation is consistent with the results of XPS analysis indicating that MIP containing ACPF is sparser and thinner than MIP without ferrocenyl monomer. The insensitivity of the current at the ACPF- MIP to VCM concentration may indicate that the ACPF-MIP is too sparse to reflect the swelling of the polymer matrix due to the interaction with the target used as a template in the permeation rate of ferrocyanide. The increase of the current at the MIP-grafted ITO with the VCM concentration shown in the DPV without the indicators (e.g., redox markers) is thought to be due to the concentration of VCM in the vicinity of the electrode surface caused by specific interaction between the MIP and VCM.

### 3.5. Response of VCM-MIP Electrode to VCM in Blood or Plasma Samples

**Figure 9:**
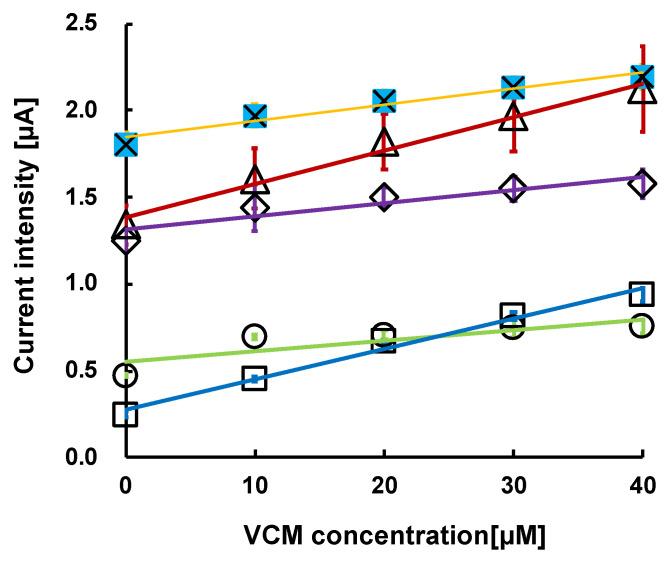
Relationship between current intensity at 0.80 V vs. Ag/AgCl with MIP grafted (including ACPF) and the VCM concentration in PBS (blue square), whole blood (red triangle), plasma (purple diamonds), washed blood cell suspension solution (green circles), and deproteinized plasma (blue squares with crosses).

The response of the VCM-MIP electrode to VCM in each of the measured solvents (PBS, whole blood, plasma, washed blood cell suspension, and deproteinized plasma) was examined. Figure 9 shows the relationship between the current value and VCM concentration in each sample solvent for VCM and Table 3 shows the background current intensity (the current detected at the VCM concentration of zero) and sensitivity.

The results showed that VCM-MIP electrodes were equally sensitive to VCM in buffered solution and whole blood. However, there was a problem in whole blood in which the current at a concentration of VCM 0 μM (background current) was higher than in the buffer solution. This phenomenon is thought to have been caused by the oxidation of concomitant components in the blood and the release of electrons, resulting in an increase in the current. Therefore, we separated whole blood into plasma and blood cells by centrifugation (3000 rpm 10 min) using 2410 (Kubota Co., Ltd., Tokyo, Japan) and used those samples to speculate which blood components were the factor that increased the background current. The obtained blood cells were re-dispersed in the PBS (0.1 M NaCl and 0.05 M sodium phosphate, pH 7.4: osmosis pressure is almost the same as blood) of the same volume as the obtained plasma to obtain a washed blood cell suspension (WBCS). Since all samples were strongly buffered at 7.4, it is unlikely that pH fluctuations affected the results.

The background current intensity in the WBCS was closest to that in the buffer solution. In contrast, the background current in plasma was almost consistent with that in whole blood. This indicates that plasma components, not blood cells, are responsible for the increase in current. However, because of the presence of proteins and other low molecular weight components in the plasma, it was not possible to identify specific blood components in that stage. Thus, a similar measurement was performed using deproteinized plasma prepared by ultrafiltration of the bovine whole blood using a hollow fiber membrane module (FB-150EGGA, NIPRO, Osaka, Japan) whose cut-off molecular weight is approximately 60,000.

**Figure 10:**
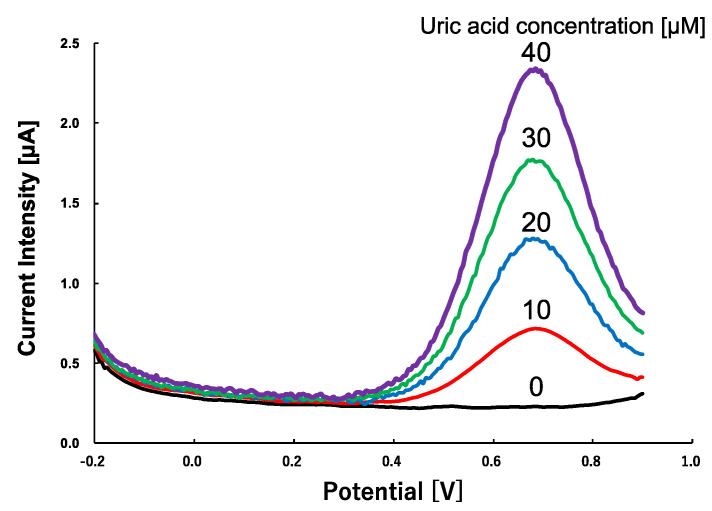
Differential pulse voltammogram of uric acid at the MIP-ITO using ACPF.

In deproteinated plasma, the current values in sections were as high as those in whole blood compared with the buffered solution. Thus, it was found that the low molecular weight component (MW < 60,000) in the blood increased the current of whole blood. This is probably because low molecular weight components in the blood can access the base electrode across the sparse MIP (using ACPF) layer and can oxidize at the base electrode. A typical low molecular weight substance that is easily oxidized in the blood is uric acid. Therefore, we obtained a differential pulse voltammogram (Figure 10) of uric acid at MIP-ITO (using ACPF) electrode and took the correlation equation between current and uric acid concentration (0–0.8 mg/dL) at 0.8 V. A linear function the of current intensity [mA] = 1.61 (uric acid concentration [mg/dL]) + 0.307 was obtained (*r*^2^ > 0.996). If the 1.1 µA difference between the current values in whole blood and buffer solution is due to uric acid, substituting this value into the equation converts the uric acid concentration in whole blood to be 0.5 mg/dL. Since the uric acid concentration in bovine whole blood is 0.71 ± 0.43 mg/dL [26], the calculated value of 0.5 mg/dL is reasonable. In other words, if the uric acid concentration in the blood is known, the background current generated from uric acid can be estimated using the calibration line of uric acid. Thus, by subtracting the calculated current intensity from the current intensity of the background current in whole blood, the calibration curve in the whole blood and that in the buffer solution can be matched. However, it is surprising that this MIP-ITO electrode showed low sensitivity in all processed blood samples (plasma, deproteinized plasma, and WBCS) while showing almost the same VCM sensitivity in the same whole blood as in the buffer solution. Since those manipulations involve slight hemolysis, it is possible that some blood cell contents may have inhibited the interaction of the VCM to the MIP. According to the results, whole blood samples should be used, when the electrode is used in actual TDM. The binding rate of VCM to albumin is usually around 55% [1], but this effect was not apparent even in whole blood in this study. This may indicate that the binding and desorption of VCM to albumin are sufficiently faster than those of VCM to MIP are.

### 3.6. Speculation of Practicality in Actual Use

Comparisons between the immunoassay of TDM [27] and the MIP-ITO electrode are shown in Table 4. The notable advantages of this electrode are its short measurement time and its ability to measure VCM concentration in whole blood. The operation time of the DPV was about 2 min. It is much shorter than the 8 min required for the operation of the immunoassay in conventional TDM. Most of the commercialized immunoassays apply optical detection, thus blood cells must be removed to prevent light scattering. More than 10-min centrifugation is required for the plasma separation [27]. MIP-ITO is expected to significantly reduce the analysis time of blood concentration for TDM. The analysis using the MIP-ITO electrode is also easy to perform since there is no need to add an indicator. Since immunoassay is a method to observe the reaction rate, it is necessary to strictly control the mixing rate and incubation time of the sample and indicator. For this reason, immunoassays require automatic analyzers, the price of which is about USD 100,000. In contrast, the price of a potentiostat required for amperometry is at most USD 10,000. Recently, TDM of VCM has been recommended to control the area under the concentration (AUC) of blood concentration over time instead of trough concentration [28,29,30] both for high therapeutically effectiveness and prevention of kidney damage. Monitoring AUC, which requires frequent analysis of the blood level of the drug, by conventional immunoassays places a heavy burden on medical staff and organizations. The MIP-ITO developed in this study enables the TDM of VCM to be performed in a point-of-care manner and simplify for analytical routine for the AUC monitoring.

However, the significant influence of reducing agents in the blood is a drawback that should not be overlooked. It is probably possible to correct the current by measuring uric acid concentration simultaneously, but the operation is a somewhat heavy burden. On the other hand, we have also developed a paste electrode of graphite particles whose surface is grafted with MIP of VCM [24]. The background current of the paste electrode sensor is almost unaffected by the blood components. However, this MIP-paste electrode requires about 5 min for the measurement, including the activation of the electrode. We believe that further investigation of the VCM detection principle of the MIP-ITO electrode and the MIP-carbon paste electrode will lead to developing an ideal sensor suitable for TDM.

## 4. Conclusions

The ITO electrode grafted with MIP using MAA and ACPF is capable of molecular discrimination to distinguish VCM from teicoplanin. In addition, the VCM-MIP electrode is highly sensitive to VCM in whole blood. The MIP-grafted electrode is feasible as a sensor for TDM of vancomycin. However, correction of the background current due to the low molecular weight reducer in the blood is required in actual clinical use.

## Figures and Tables

**Table 1:** Sensitivity to VCM or TEIC at each electrode.

Ferrocenyl Monomer	Sensitivity [mA/M]
None	0.5 ± 0.0
ACPF	17.4 ± 0.6
VF	6.7 ± 0.4
FMMA	0.1 ± 0.0

**Table 2:** Sensitivity to VCM or TEIC at each electrode.

Ferrocenyl Monomer	Electrode	Sensitivity [mA/M]		Ratio of Sensitivity (VCM/TEIC) [−]
		VCM	TEIC	
	Unmodified	4.5 ± 0.5	11.5 ± 1.1	0.4
ACPF	NIP	12.5 ± 0.8	9.6 ± 1.7	1.3
	MIP	17.4 ± 0.6	5.8 ± 0.6	3.0
VF	NIP	4.3 ± 0.5	4.2 ± 0.2	1.0
	MIP	6.7 ± 0.4	6.1 ± 0.3	1.1

**Table 3:** Sensitivity to VCM and the background current intensity at the MIP-ITO electrode (including ACPF) in each solvent.

Solvent	Sensitivity [mA/M]	Background Current Intensity [µA]
Whole blood	19.2 ± 1.9	1.34 ± 0.11
PBS	17.4 ± 0.6	0.25 ± 0.02
Plasma	7.6 ± 0.4	1.31 ± 0.06
Washed blood cell suspension (WBCS)	6.2 ± 0.2	0.55 ± 0.01
Deprotainated plasma	9.3 ± 0.2	1.84 ± 0.02

**Table 4:** Comparison between the MIP-ITO and the conventional assay for TDM.

	Detection	Operation Time	Measurement Range	Blood Cell Removal	Indicator Addition	Incubation	Interference of Redox Species in Blood	Reference
Nanopia TDM vancomycin^®^ (Sekisui Chemical Co., Ltd., Tokyo, Japan)	Optical	8 min (20 min including centrifugation)	2.5–100 μg/mL	Required	Required	Required	No	[27]
MIP-ITO electrode	Amperometry	2 min	15–60 μg/mL	Not required	Not required	Not required	Yes	This paper
MIP-carbon paste electrode	Amperometry	5 min	15–60 μg/mL	Not required	Not required	Not required	No	[24]

## Data Availability

Not applicable.
